# A study on patient perceived value in cardiology private health care services: a marketing perspective

**DOI:** 10.25122/jml-2026-0038

**Published:** 2026-03

**Authors:** Cristina Maria Soare, Traian Ioan Soare, Iuliana-Raluca Gheorghe, Florentina Gherghiceanu, Victor-Lorin Purcărea

**Affiliations:** 1 Department of Marketing and Medical Technology, Carol Davila University of Medicine and Pharmacy, Bucharest, Romania

**Keywords:** perceived value, cardiology services, health care consumer behavior, organizational image, perceived service quality, perceived service outcome, social responsibility, non-monetary costs

## Abstract

Improving service interaction and patient satisfaction requires understanding how they perceive the value of health care services. Using Weinstein's adapted Perceived Value framework, this study investigated how patients' socio-demographic factors affect their perceived value of cardiology private health care services. The perceived value variable was computed as the difference between the sum of perceived service quality, perceived service outcome, and organizational image, and the non-monetary costs. The sample comprised 210 cardiology patients, and data were collected via a self-administered questionnaire. The Mann–Whitney and Kruskal–Wallis statistical tests were used to identify socio-demographic differences, and Spearman correlations were used to assess associations. Findings revealed that social responsibility and non-monetary costs were evaluated lower than empathy, perceived service quality, and safety. Gender and education level showed statistically significant differences in the variables: performance, empathy, and non-monetary costs, respectively. The study also provides useful marketing strategies for enhancing patient-perceived value based on their socio-demographic characteristics.

## Introduction

It has been acknowledged that evaluating the effectiveness of health care services is challenging [1]. Health care services are more difficult to evaluate and manage than tangible goods because of their intangibility, heterogeneity, inseparability of production and consumption, and uncertainty about outcomes [2-4], as well as their differences from other services [5]. These characteristics often lead to discrepancies between clinical quality indicators and patients’ subjective experiences [6]. In this regard, perceived value has become an essential concept in health care marketing [7]. According to Zeithaml *et al*. [8] and Weinstein [9], perceived value is a person’s overall evaluation of the benefits gained relative to the sacrifices made after the consumption of a service, considering not only monetary costs, but also non-monetary costs, such as emotional, temporal, and psychological costs [10]. As such, the perceived value of patients plays an even more important role in influencing their health care decisions and behaviors as medical organizations increasingly embrace patient-centered approaches [11].

In cardiology services, where patients frequently face life-threatening conditions, complex diagnostic and therapeutic procedures, and long-term treatment, the significance of perceived value becomes highly relevant [12,13]. Patients’ assessments of service quality, provider competence, and technical capabilities may significantly influence treatment adherence, health care services utilization, and provider selection [14].

Although there are many dedicated scales to measure perceived value, such as the PERVAL scale [15] and Patient Perceived Value scale [16], most of them were applied in Asian health care contexts (i.e., China), leading to limited perceived value scale selection for European health care contexts [17]. Weinstein’s Perceived Value framework, also known as the S-Q-I-P framework (Service-Quality-Image-Price), reveals that perceived value is the difference between the sum of service, quality, and image, and price [9,18], offering the possibility of insights from patients and organizational performance. Weinstein conceptualized perceived value as a balance between perceived gains and perceived sacrifices and offered a thorough framework for comprehending it. According to this framework, benefits are represented by perceived service quality, perceived service outcomes, and corporate image, whereas sacrifices are primarily reflected in costs. Adapting Weinstein’s Perceived Value framework to the health care services context, the variables of interest become perceived service quality, perceived service outcome, organizational image, and non-monetary costs.

Due to its subjective nature, perceived value may differ across socio-demographic groups in the health care field [19]. According to prior studies, patients’ expectations, perceptions of risk, information processing, and perceived quality may be influenced by factors such as age, gender, and educational levels [20, 21]. For instance, studies show that younger patients may be more concerned with accessibility, information availability, and technology quality. In contrast, elderly patients are more likely to prioritize interpersonal aspects of treatment and trust in doctors [20, 21]. In a similar vein, variations in expectations, information-seeking behavior, and the assessment of service advantages versus costs have all been linked to differences in education levels [22]. In case of health care services assessment, gender differences have also been noted, as follows: female patients frequently exhibit greater sensitivity to communication quality, empathy, and emotional support, while male patients may place a larger priority on efficiency and clinical results [23]; further supporting the subjective and multifaceted nature of perceived value in health care contexts is the finding that socio-economic status affects patients’ perceptions of affordability, accessibility, and overall service value [5,8].

Despite the existing literature, insufficient research has been conducted on the factors influencing perceived value in cardiology services from a marketing perspective. The majority of current research has not explicitly examined perceived value as a multidimensional concept shaped by socio-demographic variables; instead, it has focused on overall service quality [24], patient satisfaction [25], or health care usage trends without any reference to marketing [26]. Thus, the study aimed to apply Weinstein’s adapted perceived value framework to cardiology services in order to address this research gap. More specifically, the study aimed to determine whether perceived quality, organizational image, perceived service outcomes, non-monetary costs, and perceived value differ across socio-demographic groups of cardiology patients, including gender, marital status, and educational level.

## Material and methods

### Study design and participants

The study design was cross-sectional, and participants were patients of a private cardiology healthcare organization recruited through purposive sampling. The study was conducted during March–April 2025.

Participants were provided with an envelope containing two documents: a pre-defined informed consent form and the data collection questionnaire. The informed consent form detailed the study’s objectives, the voluntary nature of participation, the confidentiality of collected data, and the use of anonymized data for strictly research purposes.

Participants were eligible if they: (1) were 18 years or older, (2) had used the organization’s services at least three times, and (3) had access to the internet or social media. Patients with cognitive impairments or those unable to provide informed consent were excluded. A non-probabilistic purposive sampling method was employed to recruit participants, ensuring inclusion of individuals with relevant health care experience. Despite setting an inclusion criterion of at least three visits for patients to use the private health care organization’s services, the non-probabilistic sampling does not ensure that each member of the population has an equal chance of being selected [27].

The sample comprised 210 patients. The sample comprised 58.1% women and 41.9% men, with a mean age of 57.62 years (SD = 15.49). Among respondents, 46.2% held a university degree, and 59.5% were married.

### Instruments

Data were collected via a paper-based, self-administered questionnaire adapted from previous studies. The instrument comprised five sections: (1) socio-demographic information, (2) perceived service quality, (3) perceived organizational image, (4) perceived service outcomes, and (5) non-monetary costs. The perceived service quality comprised 3 subdimensions: empathy (3 items; Cronbach’s alpha in this study = 0.92), tangible elements (5 items; Cronbach’s alpha in this study = 0.90), and safety (2 items; Cronbach’s alpha in this study = 0.95) [28]. The perceived organizational image comprised 2 subdimensions: social responsibility (2 items; Cronbach’s alpha in this study = 0.95) and Performance (2 items; Cronbach’s alpha in this study = 0.91) [29]. Perceived service outcome has 3 items and a Cronbach alpha coefficient in this study of 0.91 [28], while the non-monetary costs variable has 5 items and a Cronbach alpha coefficient in this study of 0.91 [30]. Participants evaluated each statement on a 5-point Likert scale, ranging from 1 (*Strongly Disagree*) to 5 (*Strongly Agree*).

### Statistical analysis

Data was processed and analyzed in SPSS version 24. Checking for Common Method Bias was assessed using Harman’s single-factor test [31]. For quantitative variables, means and standard deviations were used, whereas for qualitative variables, frequencies and percentages were used. The total scores for each quantitative variable (empathy, tangible elements, safety, social responsibility, performance, perceived service outcome, and non-monetary costs), as well as the total scores for perceived service quality, perceived organizational image, and perceived value, were tested for normality using the Shapiro–Wilk test. All variables had non-normal distributions (*P* ≤ 0.05). To identify statistically significant differences in quantitative variables depending on the socio-demographic data, the Mann–Whitney U test was used for the socio-demographic variable with two categories (i.e., gender), while the Kruskal–Wallis test with post-hoc Dunn was used for the socio-demographic data with more than 2 categories (i.e., education level and marital status). A *P* value ≤ 0.05 was considered statistically significant.

## Results

Harman’s single-factor test showed that the first factor accounted for 51.06% of the total variance, slightly exceeding the recommended threshold of 50% [31]. This indicates a potential presence of Common Method Bias; however, given the limitations of Harman’s test and its conservative nature, this result does not necessarily suggest a severe threat to the statistical analysis of the findings [31].

The normality of the variables was assessed using the Shapiro–Wilk test. According to Table 1, all variables were non-normally distributed (*P* ≤ 0.001), so the non-parametric tests, Mann–Whitney U and Kruskal–Wallis, were used. Moreover, the descriptive statistical analysis of patients’ perceptions across the dimensions of Weinstein’s adapted perceived value framework revealed generally positive evaluations of the cardiology services. Empathy (M = 4.39, SD = 0.87), perceived service quality (M = 4.38, SD = 0.70), and safety (M = 4.45, SD = 0.90) received the highest evaluations, indicating that patients value empathy, service quality, and clinical safety most strongly. Tangible elements (M = 4.29, SD = 0.76), performance (M = 4.00, SD = 0.96), perceived organizational image (M = 3.88, SD = 1.01), and perceived service outcome (M = 4.33, SD = 0.87) were also positively evaluated, reflecting favorable perceptions of both functional and organizational image. In contrast, social responsibility (M = 3.75, SD = 1.22) and non-monetary costs (M = 4.25, SD = 0.83) had lower scores.

**Table 1:** The distribution and the descriptive statistics of the study variables

Variable	Shapiro-Wilk			Mean (±Standard Deviation)
	Value of the test	Degrees of freedom	*P* value	
Empathy	0.71	210	0.001	4.39 (±0.87)
Tangible elements	0.83	210	0.001	4.29 (±0.76)
Safety	0.64	210	0.001	4.45 (±0.90)
Perceived service quality	0.79	210	0.001	4.38 (±0.70)
Social responsibility	0.86	210	0.001	3.75 (±1.22)
Performance	0.87	210	0.001	4.00 (±0.96)
Perceived organizational image	0.90	210	0.001	3.88 (±1.01)
Perceived service outcome	0.74	210	0.001	4.33 (±0.87)
Non-monetary costs	0.81	210	0.001	4.25 (±0.83)

The perceived value variable was computed as follows [8]:

Perceived value = (perceived service quality + perceived organizational image + perceived service outcome) – non-monetary costs

**Figure 1:**
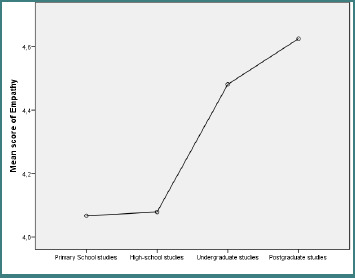
Differences in the educational level of respondents, depending on the empathy scores

**Figure 2:**
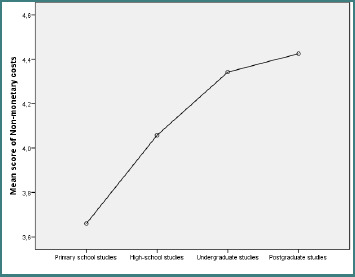
Differences in the educational levels of respondents, depending on the non-monetary costs scores

According to Table 2, a gender difference was observed in performance (Mann–Whitney U test = 4531, *P* = 0.04), with male participants scoring higher than female respondents (115.04 vs. 98.64). Table 3 showed no differences in the variables across respondents’ marital statuses. In addition, Table 4 showed significant statistical differences for empathy (Kruskal–Wallis = 15.73, *P* = 0.001) and non-monetary costs (Kruskal–Wallis = 14.23, *P* = 0.003) across education levels. The Dunn post-hoc test revealed that the statistically significant differences were registered at the empathy level between the respondents with high-school studies and postgraduate studies (4.08 vs. 4.63) (Figure 1) and at the level of non-monetary costs between the respondents with primary school studies and postgraduate studies (3.66 vs. 4.43) (Figure 2).

**Table 2:** The Mann–Whitney U tests, which depended on the gender of the respondents

	Mann–Whitney U test values	*P* value
Empathy	4944	0.30
Tangible elements	4630	0.08
Safety	5137.5	0.55
Perceived service quality	5313.5	0.90
Social responsibility	5276.5	0.83
Performance	4531	0.04
Perceived organizational image	4963	0.34
Perceived service outcome	5259	0.79
Non-monetary costs	5278	0.83
Perceived value	5036	0.44

**Table 3:** The Kruskal–Wallis test, which depended on the marital status of the respondents

	Kruskal–Wallis test values	Degrees of Freedom	*P* value
Empathy	7.47	3	0.058
Tangible elements	3.84	3	0.27
Safety	3.19	3	0.36
Perceived service quality	6.59	3	0.08
Social responsibility	4.21	3	0.23
Performance	6.90	3	0.07
Perceived organizational image	5.23	3	0.15
Perceived service outcome	5.82	3	0.12
Non-monetary costs	7.73	3	0.052
Perceived value	5.12	3	0.16

**Table 4:** The Kruskal–Wallis test, which depended on the education level of the respondents

	Kruskal–Wallis test values	Degrees of Freedom	*P* value
Empathy	15.73	3	0.001
Tangible elements	1.85	3	0.60
Safety	3.74	3	0.29
Perceived quality	4.83	3	0.18
Social responsibility	2.49	3	0.47
Performance	6.58	3	0.08
Perceived organizational image	3.85	3	0.27
Perceived service outcome	3.44	3	0.32
Non-monetary costs	14.23	3	0.003
Perceived value	1.27	3	0.73

According to Spearman’s coefficients, the strongest statistically significant correlations were observed between perceived value and perceived service outcome (*ρ* = 0.83, *P* = 0.001), performance (*ρ* = 0.78, *P* = 0.001), and social responsibility (*ρ* = 0.75, *P* = 0.001).

## Discussion

Using Weinstein’s adapted Perceived Value framework, the current study offers thorough insights into patients’ perceptions of the value of cardiology services. Overall, patients highly evaluated the perceived service quality (M = 4.38, SD = 0.70), safety (M = 4.45, SD = 0.90), and empathy (M = 4.39, SD = 0.87), suggesting that the most important features of health care services, especially in cardiology, are clinical outcomes and safety, and empathy. These findings are consistent with earlier studies emphasizing the importance of clinical competence and relational care in determining perceived value in health care settings [20,21]. Furthermore, the favorable evaluations for tangible elements (M = 4.29, SD = 0.76), performance (M = 4.00, SD = 0.96), perceived organizational image (M = 3.88, SD = 1.01), and perceived service outcome (M = 4.33, SD = 0.87) indicate that organizational image and functional quality also significantly influenced the perception of overall value [5,23]. At the same time, patients were less sensitive to the health care organization’s societal engagement or indirect effort costs, as indicated by lower scores in social responsibility (M = 3.75, SD = 1.22) and non-monetary costs (M = 4.25, SD = 0.83). This is consistent with research showing that immediate care outcomes often dominate perceptions of perceived value over broader social responsibility initiatives [32]. Thus, Weinstein’s adapted Perceived Value is multifaceted and can be applied in health care, especially in cardiology.

Differences in socio-demographic characteristics yield more significant insights. In line with other research showing gender-specific differences in health care evaluations, our study found that gender influenced performance evaluation (Mann–Whitney = 4351, *P* = 0.04), indicating that male patients may evaluate the operational efficiency of health care services more than female patients [23]. Marital status did not significantly influence perceived value in cardiology services, but empathy (Kruskal–Wallis = 15.73, *P* = 0.001) and non-monetary costs (Kruskal–Wallis = 14.23, *P* = 0.003) were strongly influenced by education level, with the postgraduate group evaluating both dimensions higher than lower-educated groups. According to earlier studies [20,21], patients with higher levels of education have higher expectations from the health care interactions. Additionally, the study findings emphasize the importance of patient segmentation strategies. Educational campaigns and service design can be customized based on patient profiles and socio-demographic differences. For instance, patients with higher levels of education may benefit from more personalized communication, such as compassionate care and assistance with service navigation. In comparison, patients with lower levels of education may place greater value on procedural guidance, clarity, and simplicity [20,21].

Strong positive correlations were found between the perceived value and perceived service outcome (*ρ* = 0.83, *P* = 0.001), performance (*ρ* = 0.78, *P* = 0.001), and social responsibility (*ρ* = 0.75, *P* = 0.001). These findings reveal that perceived value is shaped by a range of factors, with clinical outcomes and performance particularly important in high-risk, technologically demanding specialties such as cardiology [4]. According to the service-dominant logic in marketing theory, value is co-created by both functional and relational elements [33]. These results imply that cardiology service providers can maximize patient-centered value from a marketing perspective by prioritizing improvements in service outcomes and performance. To enhance patients’ trust in the clinical efficacy of care, health care organizations should invest in quality control procedures and technological innovations [4].

The study also emphasizes the strategic importance of including a social responsibility variable in health care advertising. Although social responsibility scored lower than the clinical and relational dimensions, its predictive contribution to perceived value suggests that CSR initiatives, such as sustainability practices or community health programs, may indirectly improve patient loyalty and organizational image [22]. In competitive health care markets, experts can use social responsibility communications to differentiate services, build a reputational advantage, and reinforce a comprehensive value proposition that extends beyond clinical outcomes.

### Limitations

While the study’s findings offer several contributions, they should be interpreted with careful consideration. Firstly, the study used purposive, non-probabilistic sampling, which may limit the applicability of the findings to various cardiac service populations or health care environments [27]. This sampling method may also lead to selection bias and volunteer (self-selection) bias, as noted in research on how non-random participation can skew population representativeness [34]. Secondly, self-administered questionnaires were used to collect data, which raised the possibility of response bias, social desirability effects, or participant misinterpretation of items [31], or of response bias due to the health care setting, under the “white coat effect” [35]. Thirdly, the study concentrated on the medical specialty of cardiology. While this focus provides in-depth insights, it may not accurately capture perceived value in other medical contexts, such as primary care.

### Future research directions

Building on the findings of this study, future research could take several approaches to advance our understanding of perceived value in health care services. To investigate how patients’ perceptions vary over time, especially in response to shifts in organizational initiatives, technology adoption, or service quality, longitudinal studies should be conducted [36]. A more thorough understanding of Weinstein’s adapted perceived value framework across health care contexts could be achieved by extending the research to include more patient-centered specialties, such as oncology, ophthalmology, obstetrics-gynecology, primary care, and psychiatry. Furthermore, including more complex socio-demographic factors, such as income, ethnicity, or cultural background, may help identify additional marketing segmentation methods and improve existing health care marketing strategies [5]. To provide deeper insights into patient expectations, motivations, and perceptions that standardized questionnaires may not fully capture, future research could incorporate qualitative methods, such as focus groups and interviews [22].

## Conclusion

The findings of this study revealed that perceived value should also be considered in health care marketing, as it can make significant contributions to the existing literature on patient behavior. Weinstein’s adapted Perceived Value framework can be efficiently applied in cardiology services. In addition, gender analysis emphasized the importance of different performance perceptions. In contrast, the education-level analysis suggested that differences in empathy and non-monetary costs should be taken into account, especially in segmentation methods and marketing campaigns.
